# Bridging the generational gap between nurses and nurse managers: a qualitative study from Qatar

**DOI:** 10.1186/s12912-024-02296-y

**Published:** 2024-09-05

**Authors:** Ahmad A. Abujaber, Abdulqadir J. Nashwan, Mark D. Santos, Nabeel F. Al-Lobaney, Rejo G. Mathew, Jamsheer P. Alikutty, Jibin Kunjavara, Albara M. Alomari

**Affiliations:** 1 Department of Nursing, Hazm Mebaireek General Hospital, Hamad Medical Corporation, Doha, Qatar; 2 University of Doha for Science & Technology, P.O. Box 3050, Doha, Qatar; 3 Nursing and Midwifery Research Department, Hamad Medical Corporation, Doha, Qatar

**Keywords:** Generational gap, Nursing leadership, Multigenerational workforce, Workplace communication, Nurse manager relationships

## Abstract

**Background:**

The nursing workforce comprises multiple generations, each with unique values, beliefs, and expectations that can influence communication, work ethic, and professional relationships. In Qatar, the generational gap between nurses and nurse managers poses challenges to effective communication and teamwork, impacting job satisfaction and patient outcomes.

**Aim:**

This study investigates the generational gap between nurses and nurse managers in Qatar, aiming to identify strategies to enhance collaboration and create a positive work environment.

**Methods:**

A qualitative research design was used, involving semi-structured interviews with 20 participants, including frontline nurses and senior nurse managers. Participants were purposively sampled to represent different generations. Data were collected through face-to-face and virtual interviews, then transcribed and thematically analyzed.

**Findings:**

Four key themes emerged: *Optimizing the Work Environment*: Older generations preferred transformational and situational leadership, while younger nurses valued respect, teamwork, accountability, and professionalism. *Strengthening Work Atmosphere through Communication and values*: Older nurses favored face-to-face communication, while younger nurses preferred digital tools. *Cultivating Respect and Empathy*: Younger nurses emphasized fairness in assignments and promotions, while older nurses focused on empathy and understanding. *Dynamic Enhancement of Healthcare Systems*: Younger nurses were more adaptable to technology and professional development, while older nurses prioritized clinical care and patient outcomes.

**Conclusion:**

The study reveals significant generational differences in leadership preferences, communication styles, and adaptability to technology. Addressing these gaps through effective leadership, ongoing education, and open communication can improve job satisfaction and patient care.

**Supplementary Information:**

The online version contains supplementary material available at 10.1186/s12912-024-02296-y.

## Introduction

The nursing profession faces a significant challenge of a multigenerational workforce that can cause conflict and hinder effective communication, especially between nurse managers and nurses [1]. In addition, a literature review of studies conducted over the past two decades indicates that the generational gap between nurses and nurse managers is a complex phenomenon requiring concerted efforts to address it [2, 3].

The nursing workforce comprises four generations, including the Baby Boomers (born between 1946 and 1964), Generation X (born between 1965 and 1979), Generation Y or Millennials (born between 1980 and 1994), and Generation Z (born after 1995) [4]. These generations have unique values, beliefs, attitudes, and expectations that influence their communication style, work ethic, and approach to work [4].

In 2013, Hendricks and Cope discussed the impact of generational differences on the nursing workforce and the challenges it presents for nurse managers [5]. They searched various databases electronically and found that generational diversity affects nurses’ attitudes, beliefs, work habits, and expectations. The paper suggested that accepting and embracing this diversity can lead to a more harmonious work environment and facilitate nurse retention [5].

The article focused on the cultural and work ethic differences between Baby Boomers and Generation Xers, with Baby Boomers primarily managing the workforce [6]. Baby Boomers are described as driven and dedicated, equating work with self-worth and personal fulfillment [6]. At the same time, Generation Xers have ideas of an acceptable workplace, and their terms of employment are usually non-negotiable [6]. The article summarized recent literature and studies to guide healthcare leadership in recruiting, retaining, and managing Generation X workers in the nursing field [6].

Similarly, Carver & Candela (2008) conducted a study to inform nurse managers about the generational differences among nurses and how they affect the work environment [7]. With four generations in the nursing workforce, understanding the characteristics of each generation can lead to increased job satisfaction, productivity, and decreased turnover [7]. Considering generational differences as part of an overall strategy to increase organizational commitment can improve nursing work environments and address the global nursing shortage [7]. Managers should increase their knowledge of generational diversity to tap into the strengths of each generation [7]. In addition, Younger nurses have different career expectations than their older colleagues [8]. They seek a balanced lifestyle with reasonable work hours, demand to use the latest technology, and expect to be vocal team members [8].

Managing a multigenerational workforce requires recognizing and valuing the strengths of each generation. Leaders who maximize everyone’s talents and address individual and generational needs can create synergy and improve team performance. Each generation brings unique strengths to the workforce that should be celebrated and utilized to the organization’s advantage. Meeting the needs of each employee, such as providing opportunities for advancement, work/life balance, compensation, benefits, and learning and development, can lead to higher-functioning work teams [9]. Nurse leaders should know their employees’ multigenerational characteristics and expectations and provide timely and specific feedback to manage them effectively [9]. With an appreciation of multigenerational differences and a commitment to higher-functioning work teams, leaders can improve organizational efficiency and patient care outcomes [9].

To bridge the generational gap in nursing, the SIT offers a comprehensive approach to enhancing communication, collaboration, and teamwork between nurses and nurse managers [5]. This involves acknowledging and respecting each generation’s unique characteristics, values, and experiences, which fosters a better understanding and more effective cooperation. Establishing a shared vision and goal for patient care unites nurses and nurse managers, helping to overcome any multigenerational conflicts that might arise in the workplace [5]. Additionally, encouraging multigenerational communication and mentoring is vital. This can be facilitated through programs where experienced nurses share their knowledge and skills with younger colleagues, promoting a cohesive and supportive team environment. Furthermore, providing training and development opportunities tailored to each generation’s diverse learning styles and preferences is essential for building a more skilled and competent workforce [10].

The literature indicates that the generational gap between nurses and nurse managers is a global complex phenomenon that can affect communication, work values, job satisfaction, retention, and quality of care [11]. Nursing leaders can recognize generational differences in values and behaviors as potential strengths. By gaining a deeper understanding of generational influences, these insights can be harnessed to develop effective strategies that sustain the diverse yet shrinking nursing workforce. Leveraging generational differences can also create positive work environments, enhance quality and productivity, and ultimately improve patient care. As generational differences increasingly become a critical aspect of diversity, it is essential to understand the dynamics between work engagement and meaningful work across generational cohorts to tailor approaches that align with each organization’s unique needs [12, 13].

Understanding how to bridge the generational gap in nursing is crucial for nurses and nurse managers to work together effectively and provide better patient care, ultimately leading to improved patient outcomes. This study aims to enhance workplace communication and collaboration by identifying and addressing the factors contributing to multigenerational workplace conflicts. By doing so, nurses and nurse managers can build more cohesive and supportive teams, resulting in a more positive work environment. Finally, addressing the generational gap in nursing benefits the workplace and enables the organization to develop a more engaged and motivated workforce. Multigenerational learning and development opportunities can increase job satisfaction and retention. Recognizing and valuing the unique perspectives and experiences each generation brings is essential.

### Study significance

To the best of our knowledge, no studies have been conducted in Qatar that addressed the generational gap among nurses. In line with this, the study aims to identify and compare the work engagement levels and managerial approaches among nurses and nurse managers across different generations and explore and propose effective strategies for improving communication, collaboration, and job contentment in an intergenerational work environment. The findings will contribute to the nursing profession’s knowledge and provide practical solutions for managing a diverse nursing workforce in Qatar.

## Methods

This study utilized a descriptive qualitative research design. After considering the participants’ time limits, commitments, and convenience, data were collected through semi-structured interviews with nurses and nurse managers (Executive and assistant executive directors of nursing). The authors developed the interview questions for this study (Supplementary File 1). Participants were recruited from healthcare facilities within the organization through purposive sampling. The sample size was determined based on the data saturation point, where no new themes or perspectives emerged. Interviews were conducted face-to-face or virtually, depending on the participant’s preference and availability. With the participant’s permission, interviews were audio-recorded to aid in accurate transcription and were thematically analyzed.

### Development of the interview guide

The interview guide was thoughtfully developed to capture participants’ experiences and insights effectively. The process began with an in-depth review of studies examining the generational gap between nurses and managers, identifying key themes such as work engagement, organizational environment, communication, and technological advancement. These themes provided the framework for creating open-ended questions to elicit detailed and reflective responses. Probing questions were also included to deepen the data collected by clarifying and expanding on participants’ initial answers. The draft questions underwent multiple rounds of review and refinement to ensure clarity, relevance, and the elimination of bias, with potential input from qualitative research experts.

Qualitative research aimed to generate a deep understanding of the generational gap between nurses and their managers. This understanding could not be answered in a quantitative approach. Several strategies were employed throughout the research process to ensure the credibility of the findings.

Firstly, to ensure the credibility of the data collected, the researcher established trust and rapport with the participants. This was achieved by being transparent about the research aims, building rapport, and showing genuine interest in the participants’ experiences. The researcher also ensured that the participants felt comfortable sharing their experiences and opinions by creating a safe and non-judgmental environment.

Secondly, data triangulation was used to enhance the credibility of the data. Data triangulation involves using multiple data sources to provide a more comprehensive understanding of the phenomenon being studied.

Thirdly, the researcher conducted member checking to validate the data collected. Member checking involved sharing the findings with the participants and asking for their feedback on whether the findings accurately represented their experiences and opinions. This process ensured that the researcher’s interpretation of the data aligned with the participants’ experiences and perceptions.

Fourthly, the researcher engaged in reflexivity throughout the research process. Reflexivity involves reflecting on the researcher’s biases, values, and assumptions that might have influenced the research process and findings. By being aware of their biases, the researcher ensured they did not influence the data collection or interpretation of the findings.

Finally, the researcher used a systematic and rigorous approach to analyze the data collected. This study used thematic analysis to identify patterns and themes in the data. The analysis was conducted using a coding scheme, and the findings were supported with quotes from the participants, enhancing the credibility of the findings.

#### Study population and setting

The participants were approached using a purposive sampling technique. A total of 20 participants were expected to join the study. All participants were approached based on an email from the corporate nursing mail group. The participants of this study met the following criteria: they represented diverse generations, with 3–4 from each of the subsequent generations: Generation X (1965–1980), Generation Y (1981–1996), and Generation Z (1997–2012); they had joined HMC for at least one year; and they were willing to participate in the study.

#### Study procedures

Before conducting the study, the researcher had obtained the consent of the participants (Research Information Sheet). Interviews were done face-to-face or virtually, depending on the participants’ preferences and availability. During the interviews, conversations were audio-recorded to facilitate transcriptions of the responses, completed within 24 h of the interview, and reviewed by two study researchers. The data saturation was determined by redundancy of information is indicated when similar patterns, themes, or categories keep appearing in the data, and no new information is being uncovered during additional interviews or data collection efforts.

The richness and depth of the data collected are critical. Saturation is considered reached when the data sufficiently explores and explains the research questions and key concepts, providing a comprehensive understanding of the phenomenon. Data saturation was reached after twenty interviews; however, two additional interviews were conducted to confirm this. Ethical principles were strictly observed, primarily explaining the nature and purpose of the study before obtaining their consent to participate. Identifiers were removed from the transcripts, and codes were used to label participants (e.g., Participants 1, 2, etc.). Participants were informed that they had the right to withdraw from the study at any time should they decide not to participate in further sessions.

#### Data analysis

Initially, all interviews were professionally transcribed verbatim, with pseudonyms used to anonymize participants and protect their identities. Both authors (JK and NFA) thoroughly read and re-read the transcripts multiple times to become familiar with the content and ensure the transcripts accurately reflected the audio recordings. then applied an inductive coding approach, deriving codes directly from the data rather than imposing them beforehand. This involved systematically identifying and highlighting significant quotes and segments within the transcripts that were relevant to the research questions. These initial codes were subsequently organized into potential themes by grouping together codes that shared a common essence or underlying concept. Following this, the researchers organized these initial codes into potential themes by grouping codes that shared a common essence or underlying concept.

The potential themes underwent a two-phase review and refinement process. In the first phase, the researchers reviewed the coded data extracts to ensure they coherently supported the identified themes. In the second phase, the themes were examined in relation to the entire data set to confirm that they accurately represented the data and captured the full range of participants’ experiences. Some themes were modified, combined, or discarded during this process based on their relevance and data representation.

The final step involved crafting a coherent and compelling narrative that provided a detailed account of each theme. The report included illustrative quotes from participants to substantiate the themes and vividly depict their experiences. This structured approach ensured that the analysis was thorough and that the resulting themes were deeply rooted in the data. By following Braun and Clarke’s six-step process, the study moved from raw transcripts to well-defined themes that offer meaningful insights into the generational gap among nurses and Nurse managers.

## Findings

This study had a cohort of ten frontline nurses from the new generation and ten senior nurse managers from the old generation, as shown in Table 1. The mean age of the new generation was 32.4 years (SD 4.9 years). The nurses had an average of 8.3 years of overall work experience (SD 3.09 years), specifically at Hamad Medical Corporation (HMC); they had a mean work experience of 4.7 years (SD 1.1 years). Gender distribution among the participants was 80% male and 20% female. This demographic profile reveals a well-experienced group, particularly regarding their tenure at HMC, providing a stable basis for analyzing their professional perspectives and experiences.

**Table 1:** Demographic profile for the new generation

Samples	Generation	Gender	Position	Experience in HMC(Years)	Total Experience in Nursing (Years)
P-1	Millennials	Male	Staff Nurse	5	9
P-2	Millennials	Male	Staff Nurse	6	13
P-3	Millennials	Male	Staff Nurse	5	10
P-4	Generation Z	Male	Staff Nurse	3 Months	3 Months
P-5	Generation Z	Male	Staff Nurse	3 Months	3 Months
P-6	Millennials	Male	Staff Nurse	6	10
P-7	Millennials	Male	Staff Nurse	6	9
P-8	Millennials	Male	Staff Nurse	4	9
P-9	Millennials	Female	Staff Nurse	4	8
P-10	Millennials	Female	Staff Nurse	5	9

**Fig. 1:**
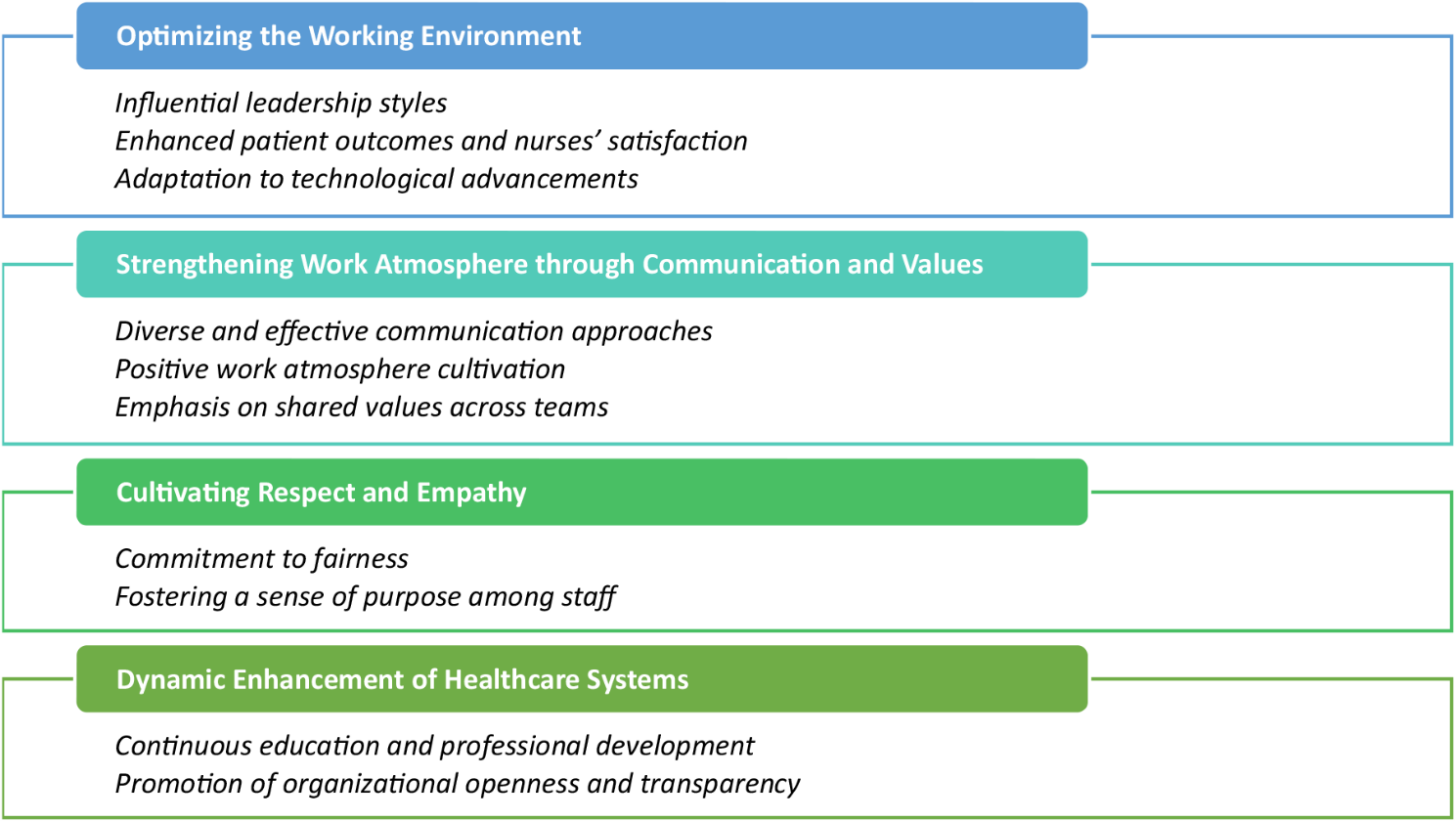
The major themes and Sub-Themes derived from the study

On the other hand, the old generation demographics: 60% were Executive Directors and 40% were Assistant Executives. Most participants belonged to Generation X (ages 44 to 59 years old), suggesting a consistent age distribution. On average, the executives had 27.9 years of overall work experience (SD 9.46 years), highlighting substantial professional tenure with considerable variability. Specifically, their mean work experience at Hamad Medical Corporation (HMC) was 17.4 years (SD 8.24 years), reflecting a diverse range of service durations at this institution. The gender distribution was evenly split, with 50% male and 50% female participants. Details on the demographic data of the old generation participants are detailed in Table 2. Three major themes were derived from the study, as illustrated in Fig. 1.

**Table 2:** Demographic profile of the old generation

Samples	Generation	Gender	Position	Experience in HMC (Years)	Total Experience in Nursing (Years)
P-11	Baby Boomer	Female	Executive Director	10	43
P-12	Generation Y	Male	Asst.Exe. Director	10	20
P-13	Generation X	Male	Asst.Exe. Director	25	9
P-14	Generation X	Male	Asst.Exe. Director	23	29
P-15	Baby Boomer	Male	Executive Director	12	40
P-16	Generation X	Female	Executive Director	35	35
P-17	Generation X	Female	Executive Director	10	27
P-18	Generation X	Male	Executive Director	9	28
P-19	Generation Y	Female	Asst.Exe. Director	19	27
P-20	Generation Y	Female	Executive Director	21	21

### Optimizing the working environment

Healthy work environments that maximize the health and well-being of nurses are essential in achieving good patient and societal outcomes, as well as optimal organizational performance. This theme consisted of three sub-themes: Influencing leadership style, Patient outcome and nurse satisfaction, and Adaptation of technological advancement.

#### Influential leadership styles

When investigating the leadership style, all older generations consistently agreed to prefer the transformational one because of its capacity to inspire and motivate frontline staff. However, to respond to specific situational demands, the older generation in our study modified and combined aspects of situational and democratic leadership.

*Which type of leadership I’m following is transformational leadership. But sometimes*,* we can take that democratic leadership in some situations*,* but not all of it. We can say situational leadership at the same time. But any leadership style you will follow should be*,* I can tell*,* a combination of some practice and attitude toward your staff”. (Participant 17).*

On the other hand, the new generation perceives leadership style by retrieving the inner values of their leaders, such as respect, teamwork, accountability, and professionalism.

*“Actually*,* our leaders primarily lead by maintaining a good relationship*,* and he is making sense of decreasing the distance between the higher and lower positions. So*,* I can say that I share the same attitudes and values with my senior managers*,* but it might differ from one person to another.” ( Participant 1).*

#### Enhanced patient outcomes and nurses’ satisfaction

The older generation perceived the working environment as a motivator for enhancing patient outcomes. Mainly, they are putting serving humanity at the top of their priority, which might be achieved through creativity, collaboration, and compassion. As articulated by Participant 7,

*“I believe that exerting the best effort in one’s job demonstrates ownership and respect for the profession. Serving humanity*,* I prioritize creativity*,* collaboration*,* and compassion in my work”.*

This quote demonstrates the deep values held by this group, highlighting their strategy of combining individual achievement with a wider humanitarian influence.

The new generation views the working environment as a vital element in improving nurses’ satisfaction, considering many contributing factors, such as the current status of the global economy and the opportunities for nurses to work and move abroad. As elaborated by Participant 13,

*“I think we can see a difference between the young and the old generation*,* and I think the way they look at nursing as a profession. There is a big difference between all the new generations*,* and I can see how the old generation looks at it. The older generation is looking at ways to help people. It is a way to provide support for older people. Unfortunately*,* I think the new generation has started looking at it as a job—more than a way of helping people. And I believe there are many different reasons for this. I think about the economic status around the world*,* and the other thing that you know is that I believe the world is open nowadays for nurses to travel around. Therefore*,* it’s started becoming a job more than a profession. Unfortunately*,* that’s why people start looking at it in a completely different way*,* which is not something good.” (Participant 13).*

#### Adaptation to technological advancements

When examining the technological aspects, the older generation acknowledges the presence of the gab. Most of them believe the gap exists because they adhere to the old practices they learned previously.

*“There is a noticeable difference between the younger and older generations of nurses*,* primarily due to advancements in technology and medical knowledge. Younger nurses are often more up-to-date with the latest care techniques and medical research*,* as they can access various modern resources. Older nurses*,* however*,* may adhere to practices they learned earlier in their careers*,* which might not incorporate recent technological changes”. (Participant 16)*

On the other hand, the new generation views new technologies as an easy-to-adopt opportunity. They like to use the new potentials that come with AI. For example, the new generation is becoming more dependent on technology due to the greater benefits it provides compared to traditional approaches in terms of diagnosis and treatment.

*“Technology is a significant factor for us*,* being part of the newer generation. It’s very important in our year of nursing. We use computers*,* advanced machines*,* and electronic documentation*,* which differ from past practices.”(participant 10)*.

*“The younger generation is adapting more easily to new technologies and software*,* like using EMR for documentation. The older generation*,* who are used to manual documentation*,* find it harder to adapt to this new system in patient care. I’ve also heard that some facilities are using GPS and AI systems to assist in diagnoses and results. So*,* artificial intelligence is becoming a part of nursing*,* and younger generations are adapting more easily to it. It will take time for the older generation to adapt because they are accustomed to different practices”. (Participant 8)*

### Strengthening the work atmosphere through communication and values

Effective communication enhances working relationships and knowledge translation and reduces conflict responsible for errors, improving patient safety. This theme consisted of three sub-themes, diverse and practical communication approaches, positive work atmosphere cultivation, and emphasis on shared values across teams.

#### Diverse and effective communication approaches

The older generation emphasizes the importance of training sessions on communication skills and advanced technologies to bridge the gap with the new generation. Moreover, they believe the new generation needs to be more skilled in direct interpersonal communication.

*“Effective communication strategies that bridge generational gaps should be promoted. This could include training on communication best practices and the use of technology for older nurses and encouraging younger nurses to develop strong interpersonal skills for face-to-face interactions”. (Participant 20)*

*“The older generations*,* always think of*,* they are more of insightful*,* in terms of*,* in the meetings they will be able to translate or interpret the information much differently. And that’s how I see.”( Participants − 18)*.

According to the new generation, effective and direct communication without any mediator can enhance the work atmosphere and ease professional communication with older generations. It can help the new generation have more chances to interact with the old generation.

*“Certainly*,* open and direct communication is helpful. As previously said*,* it is crucial to have someone who can assist in communicating with my manager in my home country. Establishing a direct line of communication with my management and developing a robust professional connection without intermediaries is vital. I appreciate the older generation’s facilitation of an open-door policy*,* as it cultivates a direct and efficient communication atmosphere.” (Participant 1).*

#### Positive work atmosphere cultivation

When examining the intergenerational dynamics in the workplace, the findings indicated that differences in experience, training, and access to technology significantly impact the work environment and the level of collaboration among employees. As one participant articulated,

*“The work atmosphere impacts collaboration. I think it does impact that and impacts these differences from one generation to another. It’s not about good and bad*,* but it’s rather about the differences in the experiences*,* differences in the training*,* and differences in the work environment as well as the availability of technology. So*,* I would say that there is a difference.” (Participant 19).* However, the new generation focuses on the technological aspect and how that might affect the work atmosphere positively.

#### Emphasis on shared values across teams

Conflicts arise when older generations rely on experience while new generations prefer evidence-based practices. This affects workplace shared values.

*“For instance*,* there might be a conflict over a non-scientifically backed common practice. The older generation might argue that they’ve been doing it for years without issues. However*,* from a knowledge-based perspective*,* the practice might be incorrect. Overall*,* the older generation’s viewpoint is based on their experience*,* where they haven’t seen negative outcomes. Conversely*,* the new generation would argue based on scientific principles and current best practices. The older generation might resist changing to these new practices. So*,* conflicts like these might arise from differing viewpoints on practices and approaches.” (Participant 9)*.

The new generations perceive shared values as part of the staff-manager relationship and can’t isolate it. When the old generation leads, the staff investigates the old generation’s way of leading, which will affect the new generation’s attitudes and values. Consequently, the new generation still takes the old generation as an example to be followed. This meaning can be found in Participant 1 answers. *“Actually*,* our leaders primarily lead by maintaining a good relationship*,* and he is making sense of decreasing the distance between the higher and lower positions. So*,* I can say that I share the same attitudes and values with my senior managers*,* but it might differ from one person to another.” ( Participant 1)*.

### Cultivating respect and empathy

This theme focuses on two subthemes: commitment to fairness and fostering a sense of purpose among staff.

#### Commitment to fairness

The results of the older generation highlight the importance of fostering empathy in the workplace. Participant 20 suggests promoting understanding by encouraging the new generation to consider their colleagues’ perspectives and motivations, enhancing mutual respect and cooperation.

*“Encourage Empathy: Foster empathy among employees by encouraging them to put themselves in each other’s shoes. Encouraging individuals to consider the motivations and experiences of their colleagues can lead to better understanding” (Participant 20).*

*“They can challenge you as a leader and they can challenge each other. That’s how you build a better workplace to have a conversation*,* a clear professional conversation. If you want to build a professional conversation*,* the two respect the critiques to respect the differences. So those differences are not conflicts. Differences are differences of opinion due to the experiences everybody can brings in.”(Participants 18)*.

However, the new generation demands that older generations be more open to work-related discussions, assignments, and promotion opportunities. They believe the new generation has a greater chance to be promoted if they get a fair chance as they are equipped and well-educated. This was clear by Participant 9.“*Compared to the older generation*,* the new generation of nurses has more opportunities for service and promotion based on education. In the past*,* nurses often held diplomas or auxiliary nursing qualifications*,* with the attitude focused primarily on patient care. Now*,* there’s a trend towards having more knowledgeable nurses capable of providing advanced care”( Participant 9).*

#### Fostering a sense of purpose among staff

A sense of purpose plays a crucial role in developing cohesive nursing teams by promoting transparent communication and mutual learning, as emphasized by Participant 18.

*“The most effective way that I felt worked during this period is the mentorship*,* working closely with the people and letting them have open communication all the time*,* providing the proper support*,* and providing the platform to share the experience and knowledge while you are learning or why they are learning from*,* and this learning process will be from both. So*,* this sharing of information through a clear mentorship*,* in one way or another*,* will create a culture of mutual respect*,* and this will end with time; this is not just easy; it takes time. But eventually*,* if it is done appropriately from the beginning*,* it will formulate a more cohesive nursing team.“(Participant 18).*

The sense of purpose was more obvious among the new generation’s responses, as can be seen in Participant 7’s response: “*Our teamwork is initially built on collaboration*,* where each nurse supports and enhances the work of others.”*

### Dynamic enhancement of healthcare systems

The new generation is more adaptable to technological changes and modern healthcare systems. They often embrace new approaches and value work-life balance and a more collaborative approach to patient care. Older nurses have been exposed to a traditional healthcare system and may have had to adapt to technological changes later in their careers.

#### Continuous education and professional development

The new generation is involved in all nursing and patient care areas. They are advancing in roles such as nurse advocates and nurse researchers. So, the new generation is expanding into new fields and trying to improve the nursing career by pursuing education and professional development. In contrast, the older generation focuses more on clinical areas and patient outcomes.

*“There are more options available now*,* especially for the younger generations. Previously*,* options were limited. You would start at a hospital or a specific department and stay there. With education and different pathways*,* you can work in patient care or move into education or other areas. This variety of options makes it easier for the younger generations.” (Participant:8)*.

*“The other thing that when you are dealing with the old generation*,* you’ll find the love to be with the patient*,* patient bedside dealing with the patient day today.” (Participant:13)*.

#### Promotion of organizational openness and transparency

The old generation perceived transparency as the need for the new and old generations to openly discuss changes, address concerns, and collaboratively adapt to evolving practices, fostering a transparent and supportive environment in the nursing profession.*“Create an environment where nurses and nurse managers can openly discuss changes in the profession*,* address concerns*,* and work together to adapt” (Participant 20).*

The new generation perceives transparency as a valuable key to promoting change. Participant No. 1’s answers reveal this meaning: *“By open communication*,* that will help. Straight communication and effective communication indeed will help in preparing for the change. As I mentioned before*,* I need some help or someone to communicate with my manager in my home country. Also*,* by ensuring that there is no second person between you and your manager*,* maintain good relationships.”(Participant:1).*

## Discussion

This study assessed the generational gap between the new and the old generation. We have identified four main themes: optimizing the working environment, strengthening the work atmosphere through communication and values, cultivating respect and empathy, and dynamic enhancement of healthcare systems. Overall, the results of this study identify the generational gap between these two generations. Moreover, the findings of this research shed light on significant subthemes that highlight the evolving dynamics within the nursing profession, particularly the differences and similarities between new and old generations. The demographic data provided a clear understanding of the structure of both generations, with a notable representation of male staff nurses in the new generation and a diverse range of experiences in healthcare.

### Working environment

Perceiving the work environment was evident as a generational gap in our study; the leadership style and other subthemes were also identified. This study discovered that the older generation significantly promotes effective leadership styles, including transformational and situational leadership. These styles enhance teamwork, promote autonomy, and ensure a supportive work environment. This is consistent with the findings of Cummings et al. (2018), who highlighted that transformational leadership positively impacts nurse satisfaction and patient outcomes by fostering a supportive and communicative work environment [14]. Furthermore, situational leadership is vital for the older generation in dynamic critical care units, offering flexibility to address staff readiness levels effectively [15].

On the other hand, the new generation stressed the importance of inner values such as respect, teamwork, accountability, and professionalism rather than the leadership style of the old generation. The new generation’s focus on internal values suggests a potential shift in organizational culture that prioritizes individual integrity and an attitude of collaboration over traditional hierarchical leadership approaches. This trend indicates that future healthcare entities’ strategies may incrementally prioritize cultivating an environment where ethical behaviors, mutual respect, and collective responsibility play crucial roles in achieving organizational success. This result is consistent with another study done by Boamah et al. (2018), who found that supportive leadership practices enhance nurses’ work engagement and patient care quality, emphasizing the need for recognition and acknowledgment strategies to boost job satisfaction [16].

In addition, our study evidently shows generational differences in adaptation to technological advancements, with the new generation demonstrating a higher ability to adopt new technologies into their practice. This finding is supported by Lera et al. (2020), who noted that the new generation is more comfortable with modern digital tools and evidence-based practices​ than the old generation [17].

### Strengthening work atmosphere through communication and values

The current study has found that generational differences in communication preferences exist, with the new generation leveraging technology for more accessible communication. In contrast, the old generation prefers face-to-face interactions for clearer understanding. This aligns with the findings of Rosi et al. (2019), who noted that younger healthcare professionals are more likely to use digital communication tools, whereas the older generation favors traditional methods [18]. Effective communication strategies that bridge these generational gaps are crucial. Training on communication best practices and the use of technology for the old generation, as well as encouraging the new generation to develop strong interpersonal skills for face-to-face interactions, are crucial [19].

Regular feedback mechanisms are crucial for identifying and addressing concerns related to the work atmosphere. Boamah et al. (2018) suggest that understanding and addressing generational differences in work preferences can improve team cohesion and reduce conflicts, ultimately leading to better patient care [16]. The study participants also emphasized the importance of feedback in creating a positive work environment, consistent with the findings of Lin et al. (2021), who stressed the value of input in fostering a supportive workplace [20]. The current study found that creating a work culture where debate is encouraged, disagreements are respectful, and active listening helps build a team-oriented mindset. This finding aligns with research by Flores et al. (2023), who noted that promoting shared values and respectful communication enhances team cohesion and collaboration [21].

### Cultivating respect and empathy

The current study has found another generational gap in respect and empathy. The new generation emphasizes the importance of having fair assignments, work-related discussions, and promotion opportunities [22]. Choi et al. (2018), consistent with our study, reported that fair clinical assignments will enhance staff satisfaction, improve nurses’ working conditions, and positively impact patient outcomes [23].

Professional self-concept is crucial to staff satisfaction, retention, and well-being [24]. The sense of purpose is part of the nurse’s professional self-concept; hence, the old generation, especially the leaders, must promote staff well-being by considering their purpose and fostering an environment of mutual benefit [25]. This finding aligns with the current study, which revealed that the new generation views a sense of purpose as fundamental to their professional needs.

### Dynamic enhancement of healthcare systems

The healthcare system is generally considered a significant influence on nursing careers. Regardless of generation, the healthcare system affects nurses and healthcare providers as it is continuously changed, modified, and developed, creating new challenges and opportunities for healthcare providers.

### Continuous education and professional development

The progression of nursing practice has been significantly influenced by advancements in education and professional development, leading to a shift in roles and opportunities for nurses. The new generation, who are more adaptable to technological changes and evidence-based practices, are increasingly moving into diverse roles beyond traditional clinical settings. They are now prominent in fields such as nurse advocacy, research, and education, reflecting a broadening of the nursing profession and ultimately enhancing healthcare systems. This shift contrasts with the experiences of the older generation who have primarily focused on direct patient care within clinical environments. Recent studies support this trend. For instance, a study found that new nurses are more likely to engage in continuous education and seek roles that allow for more incredible professional growth and diversification than older nurses [26].

### Promotion of organizational openness and transparency

Our study revealed that creating an environment that promotes openness and transparency is essential for fostering effective communication and collaboration between different generations of nurses. Fostering mentorship and knowledge sharing bridges the generational gap and ensures the transmission of valuable experiences and practices. An open dialogue between nurses and nurse managers about changes in the profession, concerns, and adaptation strategies is critical for cohesive teamwork. These findings are consistent with Bragadóttir et al. (2022), which indicate that organizational transparency and open communication channels significantly enhance teamwork and job satisfaction among nursing staff [24].

## Conclusion

This study highlights the evolving dynamics within the nursing profession, focusing on generational differences and similarities. The new generation is more skillful at integrating technology and embracing diverse roles beyond traditional clinical settings, whereas the old generation brings valuable experience and historical perspectives. Effective leadership, continuous education, and open communication are critical for optimizing the work environment, enhancing nurse satisfaction, and improving patient outcomes. Bridging the generational gap through mentorship and fostering a culture of respect and empathy are essential for a cohesive and resilient healthcare system.

### Recommendations

Future research should explore strategies to effectively bridge the generational gap in nursing by integrating leadership styles, communication preferences, and technology adoption across different generations. Longitudinal studies could examine how generational dynamics evolve as new generations enter the workforce and older generations transition out, providing insights into the sustainability of organizational changes. Additionally, expanding research to diverse healthcare settings and cultural contexts would enhance the generalizability of findings. At the same time, intervention studies could test the effectiveness of tailored mentorship programs, continuous education initiatives, and organizational transparency in fostering intergenerational collaboration and improving patient care outcomes.

### Limitation

The study’s methodology, including potential sampling bias due to purposive selection, interviewer bias, and the subjective nature of data saturation, could also influence the results. Additionally, the context-specific nature of the study and the use of virtual interviews might limit the depth and transferability of the findings. Finally, time constraints may have restricted the comprehensiveness of the data collected.

### Implications for nursing management

Nurse managers should adopt a multi-faceted leadership approach, embracing both transformational and situational styles, to meet the diverse needs of a multigenerational workforce. Implementing targeted communication training and fostering an environment of respect and empathy can improve team cohesion and patient outcomes. Investing in continuous professional development and technological training will further support the integration of new and experienced nurses.

## Electronic supplementary material

Below is the link to the electronic supplementary material.

Supplementary Material 1

## Data Availability

The data that support the findings of this study are available from the corresponding author upon reasonable request.
