# Supplementary material for: Bridging the generational gap between nurses and nurse managers: a qualitative study from Qatar
Source: BMC Nurs. 2024 Sep 5;23:623. doi: 10.1186/s12912-024-02296-y (PMC11378531; doi:10.1186/s12912-024-02296-y)
Supplement: Supplementary file 1 — Supplementary Material 1 [file 12912_2024_2296_MOESM1_ESM.docx]

**Supplementary File 1. Interview Questions**

1. How do you describe your own work attitudes and values?
   1. From your experience, how do you think your attitudes and values are different/similar from the attitudes and values of younger/older generation nurses
2. How do these differences/similarities impact communication, work atmosphere, collaboration, and contentment between younger/older generation nurses and their nurse managers?
3. How do you manage and resolve conflicts arising from generational differences in your workplace?
   1. Can you describe any specific incidents or conflicts arising from generational workplace differences?
4. What strategies can bridge the generational gap between younger generation nurses and senior managers
5. In your opinion, comparing the past with the now, has the nursing profession changed?
   1. In what aspects?
   2. How has this change impacted younger/older generation nurses' and their nurse managers' work attitudes and values?
   3. How can we prepare for these changes given the generational gap between the younger/older generation nurses and nurse managers?
   4. What role do education and training play in addressing generational differences in the nursing workforce?
